# Optimising feedback provision and literacy in medical training: linking theory to practice

**DOI:** 10.3389/fmed.2026.1883679

**Published:** 2026-09-08

**Authors:** Isaac K. S. Ng, Wilson G. W. Goh, Norman H. Y. Lin, Ming Ying Gan, Desmond B. Teo, Li Feng Tan, Ying Liang Low, Marion Margaret Aw, Adrian C. L. Kee

**Affiliations:** 1 Division of Rheumatology and Allergy, Department of Medicine, National University Hospital, Singapore, Singapore; 2 Division of Infectious Diseases, Department of Medicine, Ng Teng Fong General Hospital, Singapore, Singapore; 3 Department of Cardiology, National University Heart Centre Singapore, Singapore, Singapore; 4 Department of Hematology-Oncology, National University Cancer Institute, Singapore, Singapore; 5 Integrated Medicine Programme, Alexandra Hospital, Singapore, Singapore; 6 Healthy Ageing Programme, Alexandra Hospital, Singapore, Singapore; 7 Department of Diagnostic Imaging, National University Hospital, National University Health System, Singapore, Singapore; 8 Saw Swee Hock School of Public Health, National University of Singapore, Singapore, Singapore; 9 Division of Paediatric Gastroenterology, Nutrition, Hepatology and Liver Transplantation, Department of Paediatrics, Khoo Teck Puat - National University Children's Medical Institute, National University Hospital, Singapore, Singapore; 10 Division of Respiratory & Critical Care Medicine, Department of Medicine, National University Hospital, Singapore, Singapore

**Keywords:** feedback, feedback literacy, feedback models, medical education, medical training

## Abstract

Feedback is a cornerstone of all competency-based medical education and training programmes, where learners receive feedback from preceptors on various aspects of clinical performance to attain desired outcomes. However, feedback processes are often suboptimal due to lack of training and expertise of the preceptors in effective feedback provision leading to ineffective feedback that is lacking in timeliness, substance, actionability or palatability. In addition, inadequate feedback literacy in terms of poor self-awareness, affective overlay or lack of motivation to change further limits receptivity to constructive criticisms. In this article, we provide an educational primer on optimising feedback in medical training from both perspectives of feedback provision and reception/literacy. Through a review of common barriers to feedback identified in medical education and practice, as well as pre-existing models of feedback, we developed a novel, practical “reflect-analyse-emote-support” (RAES) framework that could be used for giving feedback in medical training, which not only retains traditional principles and learner-centered structures of feedback, but also incorporates cognitive-behavioral strategies that help navigate natural intuitive, emotive resistance to constructive/negative feedback.

## Introduction

Medical training is known to be challenging, with high proficiency standards and significant breadth/depth of knowledge/skills expected of competent physicians (1). To formalise categories of medical competencies, the Accreditation Council for Graduate Medical Education (ACGME) described six core competency domains including medical knowledge, patient care, professionalism, interpersonal and communication skills, practice-based learning and improvement, and systems-based practice (2). Central to the success of medical training in both undergraduate and postgraduate settings is the regular provision of constructive feedback based on trainee performance to cultivate good understanding of strengths/weaknesses and current progress in attainment of competency milestones, with the aim of spurring meaningful actions in professional development. However, feedback processes in medical training have often been suboptimal, due to a lack of proper training in effective feedback provision (3), and inadequate emphasis on feedback literacy/receptivity on the part of the trainees (4). For example, feedback on clinical performance may be suboptimal due to lack of direct observations of trainee performance, or unconstructive in its content (e.g., being overly vague or generic, lack of actionable suggestions) or manner of delivery (5, 6). Yet, valid constructive feedback may also be poorly received by medical trainees, due to natural emotive resistance to negative appraisals (4), coupled with inadequate self-awareness/insight (7) (e.g., Dunning-Kruger effect (8), and/or motivation to change (4).

Therefore, in this narrative review, we provide an educational primer on optimising feedback in medical training from the dual perspectives of feedback provision and feedback reception/literacy. Through a literature review, we identified the key barriers to feedback, and evaluated pre-existing models of feedback used in medical education, utilising the relevant information to develop a practical “reflect-analyse-emote-support” (RAES) tool, that retains learner-centric aspects of structured feedback in traditional models, while incorporating cognitive-behavioral strategies to navigate natural intuitive/emotive resistance to change or receiving negative feedback. Finally, we discuss specific caveats to feedback processes in medical training pertaining to workplace-based performance evaluation and professional growth that warrant consideration in future studies.

## Methodology

A narrative review approach was selected as this article was intended to explore broad and qualitative areas of feedback in medical education. For the purposes of identifying articles related to barriers to feedback provision/reception in medical training, a PubMed search was undertaken on 12 August 2026, using the following search terms: “feedback” (title/abstract), “barrier” or “pitfall” (title/abstract) and “medical training”, “medical education” or “medicine” (title/abstract). In this instance, a total of 183 articles were returned, with only 13 relevant articles selected based on relevant inclusion (primary or secondary research articles related to barriers or challenges in feedback provision or reception in context of medical training) and exclusion (articles not written in English, unavailability of full text, feedback not in context of medical education with students/trainees as the feedback recipient (e.g., medical trainees providing feedback on their program)) criteria. The first and second authors (IKN and WGG) were involved in the screening of the articles, initially by reviewing the article abstracts, and then full-text for selected articles to ensure they are relevant to our study based on the aforementioned inclusion/exclusion criteria. The decision to adopt a semi-systematic approach to identify relevant articles for this section of our narrative review was to promote objectivity and breadth in elucidating barriers to feedback. Subsequently, in our discussion on the importance of feedback from both training and clinical practice standpoints, as well as appraisal of the commonly used feedback models by clinical educators, our team utilised our own library of articles with embedded perspectives as medical educators from different disciplines with varying levels of experience who are involved in undergraduate and postgraduate training.

## What is feedback and why is it important in medical training?

Feedback can be defined simply as “information provided by an agent… regarding aspects of one's performance or understanding” (9). The aim of feedback is to be instructional in helping the feedback recipient to narrow the gap between expected and current performance/understanding (9). In the realm of clinical education, Professor Jack Ende (1983) considered feedback as “information describing students’ or house officers’ performance in a given activity that is intended to guide their future performance in that same or in a related activity, (and) is a key step in the acquisition of clinical skills” (10). Similarly, Ramani and colleagues (2015) described feedback as “a regulatory mechanism where the effect of an action is fed back to modify and improve future action” (11). In our view, feedback in medical training is, fundamentally, a practicalised form of performance appraisal, both positive (reinforcing) and negative (corrective, error signaling), that has the potential to meaningfully regulate cognitive processes in clinical decision-making and behaviors in real-world practice.

Broadly, feedback can come in several forms—they may be formative (appraisal for self-improvement purposes) or summative (performance assessment/grading) (12), delivered in written or verbal format (13), and positive/reinforcing or negative/constructive in its content (13). Most of the time, medical trainees tend to be the feedback receivers and senior medical practitioners the feedback providers. However, near-peer feedback has also recently gained popularity for its purported benefits of enhanced cognitive and social congruence (5). Regardless, feedback should be conceptualised as bilateral, context-dependent dialogue between the preceptor and trainee, with an educational alliance formed for the purpose of improving performance and promoting professional development of the trainee (14).

There are many benefits of feedback in medical training and practice. For the feedback recipient, timely, constructive feedback helps to consolidate the clinical/educational encounter, track progress of learning and provide practical guidance on how to narrow the gap between actual and desired performance. For feedback providers, the process of providing objective assessments, conveying constructive comments and suggesting actionable plans for change, not only helps to improve their teaching/coaching skills as educators, but also improves their own understanding of the subject matter (i.e., the protégé phenomenon) (15).

On the flipside, in the absence of external feedback in medical training, there can be serious repercussions on trainee professional development and downstream patient safety and clinical outcomes. This is because individual self-assessments are often highly inaccurate and subject to biases and blind-spots, often resulting in either an overestimation of their competencies (the Dunning-Kruger effect) (8) or excessive insecurities about their abilities (imposter syndrome) (16). Consequently, these individuals will not be able to correct areas that need improvement, or adequately recognise and reinforce their strengths and good clinical habits.

## The two core components of effective feedback

Effective feedback essentially comprises two key aspects: it must be delivered well by the provider of feedback and assimilated well by the recipient.

In general, high-quality feedback needs to be constructive, timely, specific, balanced and actionable (13). Effective feedback delivery involves describing a specific, observed behaviour, identifying the impact of the behaviour, and providing suggested actions to improve behavior (17). Specifically, in Hattie & Timperley's seminal work “The Power of Feedback”, they suggest that feedback on observed student behaviours may include feedback about the task, processing of the task, self-regulation, and self as a person (9). They suggest that the first three forms of feedback are useful when considering trainees' performance gaps, but the last is typically counter-productive as it deflects attention from the task and makes an unhealthy association between their self-worth/identity to the task performance (9). Regarding action plan arising from the effective feedback, the authors recommend that it should answer three key questions: “where am I going?” (the eventual goal, i.e., expected performance), “how am I going?” (the process), and “where to next?” (the immediate next actionable step) (9).

On the other hand, feedback literacy governs whether feedback received translates into meaningful actions. The concept of feedback literacy was first described by Sutton in 2012, who viewed feedback reception in three separate lenses of knowing (epistemological), being (ontological) and doing (practical) (18). Its definition was then practicalised by Carless & Boud in 2018 as “the understandings, capacities and dispositions needed to make sense of information and use it to enhance work or learning strategies” (19), and summarised into four components: 1) appreciation of feedback, 2) making judgments, 3) managing affect, and 4) taking action (19).

## Barriers to feedback in medical training

There are various barriers to feedback provision and reception in clinical training (Table 1).

**Table 1:** Barriers to feedback in medical training and mitigating strategies.

Feedback Provision	
Barriers	Mitigating strategies
Lack of time	Protected time and assigned supervisors for teaching
Lack of direct interactions or firsthand observations	Adopting mini-CEX assessments Adopting ward-based assessments
Lack of confidence/familiarity or standardisation in feedback provision	Faculty development programmes with training on feedback provision, creating psychologically safe environments conducive for feedback, and simulated practice Provision of evidence-based feedback models and templates for practical usage in routine training appraisals
Lack of interest in providing feedback	Shaping institutional culture that actively encourages feedback provision, with infrastructure in place to facilitate feedback processes
Assessment fatigue	Ensuring written feedback forms are not excessively lengthy or cumbersome to complete Promoting informal verbal feedback
Suboptimal mode of feedback provision	Using blended methods of feedback that incorporates both written and verbal feedback Ensuring protected time and confidential environments for feedback provision
Suboptimal feedback environment	Setting aside time (ideally free from interruptions) and selecting a location that affords adequate privacy to meet up with the trainee to provide feedback Having objective datapoints available during the feedback session (e.g., written feedback form, test results with trainee's responses, electronic medical records with trainee-entered documentations)
Fear of offending/upsetting the learner and/or professional hierarchial/power differentials	Anonymised and multisource feedback modalities Promoting a growth mindset in medical education and training, that sees competency development as a process Flattening of professional hierarchy and encouraging bidirectional feedback at the institutional level
Feedback Literacy/Reception	
Barriers	Mitigating strategies
Perceived lack of feedback credibility (e.g., lack of provider knowledge/expertise, presence of bias or ill-intent, inconsistent standards, unrealistic expectations, incongruency with self-assessments)	Continued professional education for skills upkeeping Selecting assessors with the relevant expertise to make the correct judgment Adopting multi-source, 360-feedback Anti-bias training for feedback providers—to identify and regulate implicit bias Bi-directional feedback (the learner also has an opportunity to provide feedback on the feedback-provider) Improve learner self-assessment capabilities through post-test diction, guided reflection, metacognitive training Reviewing exemplars to appreciate expected standards of practice
Generic/vague feedback that lacks actionability	Setting clear performance targets (interim and longer term milestones), providing practical strategies to make improvements, and scheduling regular follow-ups to track progress Create supportive environment to effect change, through behavioural nudges, improving training resource availability, and cultivating habits with action triggers and social cues
Perceived criticism that threatens self-esteem/personal worth	Mindfulness training—accepting and recognising negative emotions associated with corrective feedback as natural Cognitive reframing of feedback received (appreciating the intent of feedback, de-personalising feedback)
Top-down feedback approach that does not allow recipient sharing of personal perspectives or self-assessments	Making it a habit to elicit learner perspectives first before delivering feedback Flattened hierarchy to promote bidirectional feedback, and ease of raising concerns or seeking clarifications during feedback sessions
Natural psychological defensiveness and emotional overlay that occur when receiving negative feedback	Creating a psychologically safe feedback environment, through having one-to-one feedback with emphasis on confidentiality of content shared during feedback sessions Developing trust and a healthy feedback provider-recipient relationship Ensuring feedback provider credibility (adequate knowledge/expertise, direct observations, consistency in standards, leading by example) Near-peer feedback that may improve social/cognitive congruency Incorporating empathic techniques in communicating constructive/negative feedback

Broadly, reported barriers to feedback provision include insufficient time (20–24), lack of direct interactions or firsthand observations (21, 25, 26), lack of confidence/familiarity in clinical assessments or feedback provision (24, 25), lack of standardization in feedback methods (27), lack of interest in providing feedback (20), assessment fatigue (26), suboptimal mode of feedback provision (e.g., written/retrospective instead of verbal/real-time feedback) (23, 25), suboptimal educational environment in providing feedback (24), fear of offending/upsetting the learner (leading to “vanishing feedback”) (24, 28), and professional hierarchial/power differentials (when the feedback provider is of a perceived lower clinical rank than the recipient) (29).

On the other hand, barriers to feedback literacy/reception include perceived lack of feedback credibility (e.g., lack of provider knowledge/expertise, presence of bias or ill-intent, inconsistent standards, unrealistic expectations, incongruency with self-assessments) (23, 28), generic/vague feedback that lacks actionability (23, 24, 26, 30), perceived criticism that threatens self-esteem/personal worth (23, 28, 30), unidirectional (top-down) feedback approach that does not allow recipient sharing of their point-of-view or provide feedback to the educators (23, 30), and natural psychological defensiveness and emotional overlay that occur when receiving negative feedback, especially when the feedback is not carried out in a “psychologically safe” environment or lack of readiness of the trainee to receive it (22, 23, 31–33).

## Feedback models in medical training: From traditional approaches to a novel cognitive-behavioral centric “raes” model

### Traditional feedback models

Till date, six popular feedback models have been described in medical education and training, as summarised in a recent article (34). In chronological order of their development, the models include Mary Kay Ash's feedback sandwich (1984) (35), Pendleton (1984) feedback model (36), One Minute Preceptor model (1992) (37), ALOBA model (1996) (38), Set-GO model (1997) (39), and R2C2 model (2015) (40). A seventh recently described RAISE model (2025) (41) was also incorporated. The key features and individual strengths and weaknesses of each of these models alongside our proposed RAES model are summarised in Table 2.

**Table 2:** Feedback models and their strengths/weaknesses.

Feedback Models (34)			
Model	Key description/ features	Strengths	Weaknesses
Feedback sandwich (1984) (35)	Provides feedback in the following format: positive feedback, negative/constructive feedback, then positive feedback again	Enhances feedback palatability by cushioning negative (corrective) feedback with positive (reinforcing) feedback Simple and efficient model in a busy practice	Risks overstating the “positives” in poor performance Lacks learner centricity Lacks guidance on specifics of constructive feedback provision Lacks intuitive/emotive appeal of negative feedback Lacks guidance on actionable plans for implementation
Pendleton (1984) feedback model (36)	An extension of the feedback sandwich, to incorporate self-reflection to be more learner-centric Comprises four sequential components of 1) asking the learner what went well, 2) informing the learner what went well, 3) asking the learner what can be improved, 4) informing the learner what can be improved	Encourages learner centricity by promoting reflective self-assessment Enhances feedback palatability by cushioning negative (corrective) feedback with positive (reinforcing) feedback	Lacks guidance on specifics of constructive feedback provision Lacks intuitive/emotive appeal of negative feedback Lacks guidance on actionable plans for implementation Potentially time-consuming
One Minute Preceptor model (1992) (37)	An efficient feedback model for busy ambulatory care practice assessment Describes 5 microskills: 1) get a commitment (the trainee is to articulate differential diagnoses and management plans), 2) probing for supportive evidence (assess rationale and reasoning processes), 3) teaching general rules (giving feedback on the case), 4) reinforcement (“positive”) feedback, 5) corrective (“negative”) feedback	Highly analytical approach in feedback provision Enhances feedback palatability by cushioning negative (corrective) feedback with positive (reinforcing) feedback Efficient model in busy practice	Lacks learner metacognitive self-reflection and self-assessment Lacks intuitive/emotive appeal of negative feedback Lacks guidance on actionable plans for implementation
ALOBA model (1996) (38)	A learner-centric interview type of feedback model Comprises 5 components: 1) learner self-reflection on performance and verbalising own learning objectives/agenda, 2) eliciting learner self-assessment and own ability to problem-solve, 3) educator reinforcement of theory-practice associations and provision of balanced feedback, 4) mutual discussion and shared decision-making on methods to achieve learning goals, 5) assess overall feedback reception/literacy and summarise encounter/agreed upon action plan	Encourages learner centricity by promoting reflective self-assessment Promotes emotional receptivity of learner to feedback through bidirectional dialogue and guided interview technique	Lacks guidance on specifics of constructive feedback provision Lacks guidance on actionable plans for implementation Potentially time-consuming
Set-GO model (1997) (39)	A group encounter feedback model Comprises four key aspects: 1) learner/group reflection on performance with guided prompts, 2) eliciting learner self-assessment and own ability to problem-solve, 3) group discussion on learning objectives, 4) group discussion on action plan to achieve goal	Encourages learner centricity by promoting reflective self-assessment Useful in group learning settings May enhance feedback receptivity/literacy through informal dialogue and near-peer feedback for better social/cognitive congruence	Lacks guidance on specifics of constructive feedback provision Lacks guidance on actionable plans for implementation Potentially time-consuming
R2C2 model (2015) (40)	An overall performance feedback model Includes four key components: 1) building rapport and relationship, 2) exploring reactions to performance data, 3) exploring understanding of content, and 4) coaching for performance change (setting goals, actionable plans and overcoming barriers)	Encourages learner centricity by promoting reflective self-assessment Provides guidance on actionable plans (with emphasis on shared decision-making) for implementation Emphasis on relational aspects of the preceptor-preceptee interaction helps to improve learners’ emotional receptivity of constructive feedback	Lacks guidance on specifics of constructive feedback provision Potentially time-consuming
RAISE model (2025) (41)	An overall performance feedback model Includes 5 steps: 1) Rapport building, 2) Acknowledge students’ strengths, 3) Identify aspect(s) that need improvement, 4) Share teachers’ experiences, and 5) Establish a plan to improve	Enhances feedback palatability by cushioning negative (corrective) feedback with positive (reinforcing) feedback Learner reflection is embedded within the steps Provides guidance on actionable plans (with emphasis on shared decision-making) for implementation Emphasis on relational aspects of the preceptor-preceptee interaction (e.g., rapport building, sharing of trainer's personal experiences) helps to improve learners’ emotional receptivity of constructive feedback	Lacks guidance on specifics of constructive feedback provision Learner self-assessment not explicitly requested Potentially time-consuming
Proposed RAES model	A practical model that builds upon the strengths of traditional models in promoting learner metacognition in self-reflection, offers constructive actionable feedback that is reasonable, and optimises emotional receptivity of the intuitive mind Involves four core components: reflect, analyse, emote, support	Psychological grounding in dual process theory where feedback provision strategies hinges on both presentation of objective data and factual examples, with constructive action plan to appeal to the learners’ analytical mind, and multi-pronged strategies to optimise emotional acceptance of negative/constructive feedback with adequate support in behavioral change	Not presently validated or trialed in real-world settings Potentially time-consuming

While the above feedback models have their individual merits, they are, in our view, relatively limited in terms of addressing feedback literacy, particularly in terms of psychoemotional palatability and intuitive appeal of feedback which are necessary to engender behavioural change.

### Linking cognitive theories that govern human behaviors to feedback literacy

Cognitive science research suggests that our brain operates on a dual process model (i.e., “*thinking fast and slow*”) that comprises system 1 thinking which is intuitive, fast, automatic, emotive, subconscious and more error-prone/biased, and system 2 thinking that is analytical, slow, intentional, more rational, conscious and usually more accurate (42, 43). In his earlier works on moral judgments, social psychologist Jonathan Haidt described how moral reasoning does not produce moral judgment but rather it is a *post-hoc* explanation created to rationalise an intuitive judgment (44). In his subsequent book “The Happiness Hypothesis”, Haidt conceptualised human decision-making with a metaphor of an elephant (represents intuition/system 1 thinking) and a human rider (represents analytical/system 2 thinking) (45), arguing that behavioural change is notoriously difficult because the analytical mind (the rider) is often outmuscled by emotions (the elephant) who does not want to follow where logic leads to.

We opine that this dual process cognitive model offers a useful conceptual lens through which we contemplate feedback literacy of learners. Both systems are relevant—intuitive rejection of negative feedback due to psychological overlay and analytical disagreement with feedback due to inconsistent assessment standards, lack of direct observation, perceived assessor bias, and legitimate differences in interpretation of task performance.

Therefore, effective feedback requires not only conveying the right information and providing an actionable plan that is reasonable to the logical mind, but also involves optimising its intuitive appeal and psychoemotional palatability, with cultivation of a psychologically safe/supportive environment to enact behavioral change.

### Towards a novel “Reflect-Analyse-Emote-Support” (RAES) feedback model

We herein propose a novel “Reflect-Analyse-Emote-Support” (RAES) feedback model (Tables 3,4) that builds upon the foundation of traditional feedback models, with explicit incorporation of cognitive psychology-guided framework to delivering feedback that is reasonable, actionable and palatable.

**Table 3:** A novel “RAES” feedback model.

Component	Description
Reflect	Objective: addresses learner insight/metacognition Requires learners to self-appraise performance, justify decision-making, highlight own strengths and weaknesses and come up with possible strategies to improve
Analyse	Objective: target system 2 thinking; appeal to the logical mind Facilitate learner interpretation of factual/objective performance data → eliciting implications of these findings (e.g., on professional competency, entrustability, patient outcomes) → uncovering hypotheses or reasons for the performance → coming up with practical solutions to improve
Emote	Objective: target system 1 thinking; appeal to the intuitive mind For feedback providers: seek to reduce emotive resistance to negative/constructive feedback through enhanced feedback provider credibility (ensure assessor knowledge/expertise in subject matter, due diligence in making direct performance observations, consistent assessment standards, minimise bias, and leading-by-example to avoid perceived hypocrisy), adopting near-peer teaching/feedback for improving social/cognitive congruency, building supervisor-supervisee educational alliance and incorporating empathy in feedback delivery For feedback recipients: enhancing feedback literacy through educational pedagogies built on Winstone et al.'s SAGE taxonomy framework (self-appraisal, assessment literacy, goal-setting, engagement/motivation) (54) and helping trainees adopt psychological interventions (e.g., mindfulness, cognitive-behavioral techniques) to process and regulate negative emotions associated with feedback
Support	Objective: shaping a viable path to effect change Requires creating a conducive environment for self-directed learning and trainee identity development, and adopting behavioural nudging, action triggers, and social cues to facilitate change

**Table 4:** Example of the RAES model applied to a medical training scenario.

**Vignette**: John, a final year medical student on his internal medicine rotation, is observed by his supervisor, an infectious diseases consultant, breaking the news of a newly diagnosed human immunodeficiency virus (HIV) infection to a patient in the ward. This patient was a 35-year-old gentleman who had been admitted for persistent fever of 2-week duration with no localizing symptoms/signs. After eliciting the patient's perspective on what he had been admitted for and why a HIV test was performed, John went straight to delivering the news that the HIV test came back positive, and provided appropriate and comprehensive information about the implications of a HIV diagnosis and effective antiretroviral treatments options available. However, he was noted to have delivered the information fairly abruptly without adequate prior warning shots, used excessive medical jargon, and moved quickly into discussing treatment options without exploring the patient's understanding, concerns, or noticing his emotional response. After the consultation, John's supervisor brought him to an empty staff room to discuss the encounter and fill up a mini-CEX form.	
**Component**	**How it applies to this case**
Reflect	John is first asked to reflect on the consultation: what he thought went well, what he found challenging, and how it could have been improved. He shared that he was clear in his delivery of information and provided accurate, factual medical information. On hindsight, he felt that perhaps he might have spoken too fast and should have checked whether the patient understood him at appropriate junctures.
Analyse	The supervisor affirmed that John had indeed provided accurate medical information and was comprehensive in covering the relevant information about the HIV diagnosis and available treatment options during the clinical consultation. He also agreed with John's observations that perhaps he could have slowed down a bit during the communication and pause to check for patient's understanding before moving on to the next discussion item. In provision of constructive feedback on the manner of delivery of the bad news, the supervisor provided specific observations such as “*I noticed that you relayed the diagnosis to the patient straightaway right after eliciting the patient's perspectives on why the HIV test was done. The patient appeared to be taken aback and did not respond to you much over the next few minutes as he seemed to be in shock after receiving the news. Typically, in clinical consultations that involve breaking bad news, we advise providing one or two prior warning shots so that the patient is more prepared to accept the incoming unfavorable information being delivered.”*. He then asked John if there were particular reasons why he decided to deliver the information straightaway to the patient to which he replied that he was “*not sure*”. The supervisor then offered possible explanations for John's consideration such as his relative inexperience as a medical student in breaking bad news in clinical settings or perhaps he felt that there was no need to “beat around the bush” in this context. He suggested that John could practise again with breaking similar news to another patient with newly diagnosed HIV in his infectious diseases clinic in the coming week, but this time, utilise warning shots such as “*The test results have come back. Could I share it with you now?*” and “*I'm afraid it's not good news. May I continue?*”, and observe whether if there are any differences in how the patient reacts to the news in both circumstances. In addition, the supervisor also relayed that “*I noticed you used quite a number of medical jargons such as “anti-retroviral therapies” and “opportunistic infections”—which I'm not so sure that the patient understood*”. He offered that perhaps these could be replaced with more layman terminologies such as treatment of the HIV (a viral) infection, and infections by less usual bugs that may occur when someone's immune system is compromised.
Emote	John initially appeared uncomfortable when receiving the constructive feedback and turned somewhat defensive because he felt that being clear and comprehensive constituted good communication in context of what is often considered to be an uncomfortable topic to discuss with patients. The supervisor acknowledges that breaking of bad news is a challenging task even for seasoned clinicians like himself who has done it many times. He normalises the discomfort associated with discussing a HIV diagnosis, particularly due to the stigma that could be associated with the diagnosis. He also shared his own personal experience and how he struggled during his first clinical encounter involving disclosing a new HIV diagnosis to a patient. He shared that he engaged in self-reflection after that encounter when he was a young clinician, and felt he was able to better empathise with the patient after he re-played the scene in his mind to imagine how the encounter must have felt coming from the patient's perspective, which then prompted him to improve on certain aspects of the content delivery. He encouraged John to do the same, and spend some time reflecting and putting himself in the patient's shoes after such communication encounters. He also suggested that John could try out some of the suggestions he offered earlier for the subsequent communication tasks on breaking bad news, and see for himself if they help with the conversation. The supervisor again reiterated that he sees a lot of potential in John to becoming a good clinician by virtue of having in-depth medical knowledge at final year level, and he hopes by strengthening his communication skills, he will be a more well-rounded clinician in future.
Support	The supervisor ended off by suggesting that John sits in his upcoming infectious diseases clinic and perhaps observe how he conveys HIV diagnoses to his patients, before re-attempting it with supervision. He advised John to read up on the “SPIKES” (setting up, perception, invitation, knowledge, empathy, summary) model to familiarize with this before their subsequent clinic encounter.

In this model, the four core components include: 1) learner self-reflection and self-assessment, 2) provision of learner-centric, structured feedback that appeals to the analytical mind, 3) enhancing feedback literacy and psychoemotional palatability of feedback to persuade the intuitive mind, and 4) development of an overarching supportive environment to facilitate sustained behavioural change. The novelty of this model lies in its psychological grounding in the dual process theorem where trainers explicitly adopt feedback provision strategies that involve reviewing objective data and factual examples, with constructive action plan that appear reasonable to the learners' analytical mind, and utilize multi-pronged strategies to enhance emotional acceptance of negative/constructive feedback with adequate support in behavioral change. It also retains the important components of learner metacognition through self-reflection and supporting/engendering behavioral change as advocated in prior models like ALOBA (37), SET-GO (39), and R2C2 (40).

Firstly, pre-feedback trainee self-reflection on own performance, strengths and weaknesses is highly important, as it highlights the degree of discrepancy between self-appraisal and objective assessment. If there is a significant gap in self-assessment and objective feedback identified, then targeted pedagogical modalities to enhance learner insight need to be employed—for instance, guided reflection with verbalisation of thought processes and gentle correction/challenging of incorrect narratives, metacognitive training and analysing of performance exemplars to be more attuned to expected standards (7).

Secondly, the process of feedback provision should be logical and well-reasoned, backed by supportive factual/objective evidence, in order to engage the learner's analytical mind. Broadly, feedback can be structured in the following manner: 1) identifying known/undisputed facts or objective assessment finding(s) (e.g., performance scores, time taken or errors made during a clinical task), 2) discussing the implications of the finding(s) (in relation to professional competencies, entrustability, and patient outcomes), 3) discussing hypotheses to explain performance, and 4) developing practical, actionable strategies to engender change and monitor progress. In particular, a constructivist approach of facilitative and dialectical feedback (where learners' self-reflection, perspectives and clarification are encouraged) is preferred to a traditional cognitivist framework of corrective, top-down critique (46), due to greater efficacy in promoting sustained, self-driven behavioral change.

Thirdly, feedback literacy must be enhanced through appropriate engagement of the feedback recipient's intuitive/emotive mind. This component requires targeted interventions directed at both the feedback provider and feedback recipient, because feedback literacy is ultimately governed by a “bidirectional empathy” between the feedback provider and recipient in understanding and appreciating each other's perspectives. The word “empathy” (also known as *Einfuhlung*) refers to a process of de-centering from one's context, re-imagining from the perspective of another, and approximating of observed cognitive and affective states (47). Therefore, during a feedback session, the feedback provider must be cognisant of how feedback is likely to be perceived by the recipient, and tries to frame the message in a palatable manner, sensitive to personal circumstances or contextual influences which could have affected performance (4). On the other hand, the recipient must demonstrate reciprocal empathy by metacognitive contemplation of the intent of the feedback provider, to recognise that well-meaning, constructive feedback is actually a gift to aid improvement, given the relational risks involved in the provision of corrective feedback.

On the side of feedback providers, several interventions are helpful to navigate natural intuitive/emotive resistance to constructive feedback. Firstly, perceived feedback credibility must be enhanced through selection of performance assessors with adequate knowledge/expertise of the assessed subject matter, ensuring adequate direct interaction/observations of learner performance prior to feedback provision, and regular audits on consistency of assessment standards (with safeguards and assessor training on implicit bias). In particular, having clinical supervisors practise positive role-modelling (i.e., leading by example) is extremely important, as it is intrinsically able to motivate trainees to want to emulate their actions and behaviours (48). Otherwise, feedback provided by someone who does not practise what he/she preaches will naturally be perceived as hypocrisy (49) and gets intuitively rejected. Secondly, emotional and social congruence can be enhanced through near-peer teaching/feedback (50), and prior nurturing of genuine supervisor-supervisee relationships in forming an “educational alliance” (14, 51). Thirdly, the feedback provider should actively consider the trainees' perspectives and contextual factors that could have influenced performance, and adopt the relevant empathic communication techniques to improve palatability of feedback content. These include the use of normalising statements (52) (e.g., normalising the negative emotions in receiving constructive feedback or challenges in making necessary changes), reflective statements (53) (acknowledging expressed emotional content), and/or validation/affirmation (of what has been done well or presence of commitment). Moreover, feedback should not be excessively harsh or contain character insinuations, but instead rely on factual information and emphasis of benefits of the desired improvements in achieving expressed values/training goals of the feedback recipient.

For feedback recipients, they should be educated on how to gain maximally from feedback, empathise with the intent of the feedback provider, and effectively regulate negative emotions associated with feedback. In a recent paper on feedback literacy (4), Winstone et al's SAGE taxonomy framework (54) was highlighted as an important guide for educational interventions to improve learner engagement with feedback. In brief, it comprises four components of developing self-appraisal (e.g., post-test self-assessment), fostering assessment literacy (e.g., understanding the marking rubrics), promoting goal-setting (creating goals and action plans), and enhancing learner engagement/motivation (e.g., developing an innate desire to seek out feedback to achieve stipulated goals) (54). In particular, learner metacognition/self-appraisal capabilities must be strengthened through regular self-assessment practices (33) to avoid the Dunning-Kruger effect (8). Studies have also highlighted that the practice of near-peer feedback which immerses learners in the feedback creation process is helpful to develop an understanding of the point-of-view of the feedback provider, and review of exemplars or reflective journaling can further enhance trainee self-awareness through metacognition (19, 55). In addition, psychological training can be provided to medical trainees to help them cultivate the right mindset (cognitive framing) towards feedback (33), and process and regulate negative emotions associated with constructive feedback, such as mindfulness interventions (56), or cognitive-behavioral techniques such as cognitive re-framing and objective appraisals (57). Likewise, the trainee should also be willing to build healthy relationships with their preceptors (33) in the bidirectional educational alliance (14).

Finally, a supportive and conducive environment must be created to facilitate feedback implementation and meaningful behavioural change. This can be achieved through behavioural nudges, and promoting of good habits through action triggers and social cues. For example, if a trainee physician is prone to cognitive bias such as premature diagnostic closure, then cognitive forcing or pivot-and-cluster strategies can be adopted when making important diagnostic decisions in order to ensure important or dangerous diagnoses are not missed (58, 59). In addition, the use of social cues to motivate change applies to clinicians too—where a randomised controlled trial found that a social norm feedback intervention (that highlights how one's practice deviates from the norm) substantially improved general practitioner antibiotic prescribing habits (60).

Admittedly, while this novel feedback approach was developed through incorporation of cognitive psychological principles into traditional feedback models, its efficacy in real-world clinical training remains to be seen, and further proof-of-concept studies are necessary to evaluate and validate this model.

As clinical training institutions endeavour to implement a positive feedback culture, the quality of feedback provided in a specific learning environment or from a specific trainer can be meaningfully assessed by validated instruments such as the FEEDME-Culture and FEEDME-Provider surveys completed by the feedback recipient (61). Regular audits of the strengths and weaknesses of feedback that occurs within an educational system can help to tailor interventions to optimise its delivery.

## Caveats of feedback in workplace-based assessments and professional growth

Firstly, when feedback is utilised for workplace-based summative assessments, it needs to be sufficiently holistic and unbiased—which means it should be multi-timepoint, multi-modal and multi-source (360-degree model) (1). This is because trainee performance is not always necessarily reflective of competency, as it may be subject to personal circumstances and situational variables (1). Moreover, performance assessments are subject to both intra- and inter-assessor variation, as well as bias (especially if the assessor is considered a personal mentor to the trainee) (1). As such, the adoption of anonymous, multisource feedback helps to ensure a more well-rounded and objective evaluation of trainees’ clinical and non-clinical attributes displayed in learning and professional practice. For example, while senior clinicians may be often the main assessors of clinical knowledge, acumen and skills, peers may be better placed to assess teamwork aspects, while juniors may assess teaching/supervisory skills, and nursing and allied health staff may be able to provide feedback on interprofessional collaboration, communications and collegiality.

Secondly, given that feedback for summative assessments is typically provided by officially assigned clinical supervisors, we suggest that it may be beneficial to have a healthy separation of mentorship/coaching and supervisory roles in real-world practice. This is because the authenticity of interactions between a trainee and clinical supervisor is often influenced by the desire to project the best possible image during work performance evaluations (62), preventing honest sharing of vulnerabilities and struggles faced during clinical training. This in turn impedes professional growth and development of the medical trainee.

Therefore, future studies would be helpful to evaluate the pedagogical strategies that can effectively nurture and navigate the symbiotic relationship between feedback processes in medical training, work performance assessments, and trainee growth and professional development.

## Conclusion

In summary, feedback is a quintessential aspect of medical education and training, which is necessary to attain and maintain professional competency. Effective feedback requires both constructive and timely feedback provision as well as recipient feedback literacy. In this article, we described a novel “reflect-analyse-emote-support” (RAES) model of feedback in medical training, that seeks to combine learner-centric principles of structured feedback with cognitive psychology-guided strategies that improve palatability of feedback and engender sustained behavioural change.
